# Evaluating the efficacy of a multistrain probiotic supplementation for prevention of neonatal sepsis in 0–2-month-old low birth weight infants in India—the “ProSPoNS” Study protocol for a phase III, multicentric, randomized, double-blind, placebo-controlled trial

**DOI:** 10.1186/s13063-021-05193-w

**Published:** 2021-04-01

**Authors:** Anju Pradhan Sinha, Subodh S. Gupta, Ramesh Poluru, Abhishek V. Raut, Narendra Kumar Arora, Ravindra Mohan Pandey, Aditya Ranjan Sahu, Adhisivam Bethou, Sunil Sazawal, Sailajanandan Parida, Ashish Bavdekar, Arvind Saili, Rajni Gaind, Arti Kapil, Bishan S. Garg, Chetna Maliye, Manish Jain, Kamlesh S. Mahajan, Pratibha Dhingra, Keshab C. Pradhan, Anand S. Kawade, Sushma Nangia, Ajit Mukherjee, Reeta Rasaily, Radhey Shyam Sharma, the ProSPoNS study Group, Pankaj Gupta, Amritanshu Kumar, Sukanya Sarma, Apoorva Mathur, Dixit Prajapati, Kanchan Yadav, Neeraj Kumar Kashyap

**Affiliations:** 1 Division of Reproductive Biology, Maternal & Child Health (RBM&CH), Indian Council of Medical Research (ICMR) Headquarters, V Ramalingaswami Bhawan, Ansari Nagar, New Delhi, Delhi 110029 India; 2 Department of Community Medicine, Dr. Sushila Nayar School of Public Health, Mahatma Gandhi Institute of Medical Sciences (MGIMS), Sewagram, Wardha, Maharashtra 442102 India; 3 The International Clinical Epidemiology Network (INCLEN) Trust International, F-1/5, 2nd Floor, Okhla Industrial Area Phase - 1, New Delhi, Delhi 110019 India; 4 Department of Biostatistics, All India Institute of Medical Sciences (AIIMS), New Delhi, Delhi 110029 India; 5 Next Gen Pharma India Pvt. Ltd., 331, Sector 15A, Noida, Uttar Pradesh 201301 India; 6 Department of Neonatology, Jawaharlal Institute of Postgraduate Medical Education and Research (JIPMER), Dhanvantri Nagar, Gorimedu, Puducherry 605006 India; 7 Centre for Public Health Kinetics (CPHK), 214 A, Vinoba Puri, Lajpat Nagar-II, New Delhi, Delhi 110024 India; 8 Neonatal Health & Human Nutrition, Asian Institute of Public Health (AIPH), 8A, Unit-6, Ganga Nagar (Near Raj Bhawan), Bhubaneswar, Odisha 751001 India; 9 Department of Pediatrics, KEM Hospital Research Centre, 489 Rasta Peth, Sardar Moodliar Road, Pune, Maharashtra 411011 India; 10 Department of Neonatology, Lady Hardinge Medical College and Associated Kalawati Saran Children’s Hospital (KSCH), Near Gole Market, Central Dist., New Delhi, Delhi 110001 India; 11 Department of Microbiology, Vardhaman Mahavir Medical College and Safdarjung Hospital (VMMC & SJH), Ansari Nagar (W), New Delhi, Delhi 110029 India; 12 Department of Microbiology, All India Institute of Medical Sciences (AIIMS), New Delhi, Delhi 110029 India

**Keywords:** Neonatal sepsis, Low birth weight, Probiotics, Vivomixx®, India, Randomized controlled trial, Study protocol, India

## Abstract

**Background:**

Progress has been made in the reduction of under-five mortality in India; however, neonatal mortality is reducing at a slower rate. Efforts are required to bring down neonatal mortality in order to attain the Sustainable Development Goal-3. Prevention of sepsis among the high-risk, vulnerable low birth weight neonates by a newer intervention with probiotic supplementation is promising.

**Methods:**

A phase III, multicenter, randomized, double-blind, placebo-controlled study is being conducted at six sites in India. A total of 6144 healthy low birth weight (LBW) infants fulfilling the eligibility criteria would be enrolled within the first week of life, after obtaining written informed consent from the parents of the infant. Randomization in 1:1 ratio, stratified by site, sex, and birth weight, would be done through an interactive web response system (IWRS) using a standard web browser and email service. Vivomixx®, a probiotic containing a mix of 8 strains of bacteria, in a suspension form standardized to deliver 10 billion CFU/ml, or an organoleptically similar placebo would be fed to enrolled infants in a 1-ml/day dose for 30 days. The follow-up of enrolled infants for 60 days would take place as per a pre-specified schedule for recording morbidities and outcome assessments at the six participating sites. Screening for morbidities would be conducted by trained field workers in the community, and sick infants would be referred to designated clinics/hospitals. A physician would examine the referred infants presenting with complaints and clinical signs, and blood samples would be collected from sick infants for diagnosis of neonatal sepsis by performing sepsis screen and blood culture. Appropriate treatment would be provided as per hospital protocol. The study would be implemented as per the MRC guideline for the management of Global Health Trials in accordance with ICH-GCP and Indian Regulatory guidelines. A contract research organization would be engaged for comprehensive monitoring and quality assurance. The final analysis would be conducted in a blinded manner as per the statistical analysis plan (SAP) to estimate the primary outcomes of sepsis, possible serious bacterial infection (PSBI), and secondary outcomes. The codes will be broken after DMC permission. The protocol has been reviewed by the Research Ethics Committee of the Liverpool School of Tropical Medicine (REC-LSTM), from Research Ethics Committees of the six subject recruitment participating sites.

**Discussion:**

This adequately powered and well-designed trial would conclusively answer the question whether probiotics can prevent neonatal sepsis in the high-risk group of low birth weight infants as indicated by a pilot study in 1340 LBW infants, evidence from systematic reviews of hospital-based studies, and a primary study on healthy newborns in Orissa. Results of the study would be generalizable to India and other low–middle-income countries.

**Trial registration:**

Clinical Trial Registry of India (CTRI) CTRI/2019/05/019197. Registered on 16 May 2019

## Background

India has made considerable progress in reducing the under-five mortality rate in the past decade. Tremendous efforts are being taken by the government to achieve the 3rd Sustainable Development Goal (SDG) of under-five mortality of 25 per 1000 live births by 2030. It would be attainable only if the neonatal mortality rate, the most important contributing factor for the high under-five mortality, is reduced [1, 2]. Although the neonatal mortality has declined from 38/1000 in 2000 to 23.5/1000 in 2017 [3], it is much slower than the decline in the US mortality rate observed during the same period. Any further decline in neonatal mortality would require newer modalities to prevent/manage neonatal infections (pneumonia, septicemia, meningitis) that are responsible for more than a quarter of the 1 million neonatal deaths every year in India [2].

Neonatal sepsis is accountable for a high risk of adverse long-term neurodevelopment outcomes in susceptible infants [4–6]. Low birth weight (LBW) neonates have a poor cognitive function and compromised immune functions, and LBW is an important indirect cause of death in neonates, accounting for 40 to 80% of neonatal deaths [7–9]. Infections are known to spread rapidly leading to severe disease and death in LBW neonates, and therefore, prevention of infection would directly decrease neonatal morbidity and mortality. Management of neonatal sepsis with antibiotics attributing to their easy availability over the counter, indiscriminate use, and incomplete courses has resulted in widespread drug resistance in India. Currently, there are no preventive interventions available for neonatal sepsis other than good hygiene practices and exclusive breastfeeding.

The gut microbiome is a complex and dynamic population of several hundred bacterial species known for delivering nutrients, regulating the maturation of intestinal epithelium, and developing an innate immune defense in newborns [10, 11]; however, very low birth weight (VLBW) and LBW neonates have been reported to possess delayed colonization of normal bacterial species, in addition to a much lesser microbial diversity in the intestinal tract as compared to term infants [12]. WHO defines probiotics as “live micro-organisms which when administered in adequate amounts confer a health benefit on the host” [13]. Though the exact mechanism of action of probiotics is not known, they are assumed to protect neonates from developing sepsis by improving gut barrier function, competitive exclusion of pathogens, modification of host immune responses, augmentation of immunoglobulin A (IgA) mucosal responses, and enhancement of enteral nutrition that inhibits the growth of pathogens and aids in upregulation of immune responses [14–17]. Several authors have reported a lower incidence of late-onset sepsis (LOS) with probiotic supplementation [18–20], and the positive results from these systematic reviews of hospital-based randomized controlled trials (RCTs) justify the need for a large, randomized, multicenter community study to demonstrate the efficacy of probiotics among healthy LBW infants in settings with a high burden of neonatal sepsis. Panigrahi et al. reported a significant reduction in the primary outcome (a combination of sepsis and death) in the treatment arm (risk ratio 0.60, 95% CI 0.48–0.74) receiving a symbiotic preparation (*Lactobacillus plantarum*, plus fructooligosaccharides) in a study conducted among healthy newborns, in Orissa, India [21].

An earlier study conducted by us in 1340 neonates reported a non-significant 21% reduction in the risk of potential suspected bacterial sepsis (PSBI) and a 33% overall reduction in risk of sepsis through a physician diagnosis in post hoc analyses. The analysis of an un-prespecified sub-group of infants weighing 1.5–1.99 kg showed a 100% reduction, with no sepsis cases in the probiotic arm [22]. Presently, we plan to conduct a large, multicentric, parallel-arm, placebo-controlled study using the same probiotic to conclusively evaluate the role of probiotic in the prevention of neonatal sepsis. The current study proposes to use the probiotic preparation at the same dose and regimen but in a newer, safer, and convenient dosage form.

## Methods

### Study design and study sites

This phase III, multicentric, randomized, double-blind, placebo-controlled study is supported by the UK Research and Innovation, Medical Research Council, UK, under the Global Health Trials [JGHT] scheme 8, jointly funded by the DFID/NIHR/MRC/Wellcome Trust. The study participants would be recruited from six participating sites that have been identified and selected by the trial advisory group of the Indian Council of Medical Research (ICMR), the sponsor of the trial, following a transparent procedure based on evaluation of the following criteria: experience in the conduct of community-based studies; a composite score based on publications by the site investigators in high impact journals; competence in the measurement of morbidities judged as per publications; infrastructural facilities relevant for conduct of the clinical trial; availability of recognition certificate from the Department of Science and Industrial Research, Government of India; linkages with local hospitals; and capability to recruit LBW infants in stipulated time and their geographical distribution across India. The investigators with a proven track record in the conduct of community-based research studies in maternal and child health were considered at the chosen sites. The sites selected for the study included Kalawati Saran Children’s Hospital (KSCH), New Delhi, and Center for Public Health Kinetics, New Delhi (with recruitment sites at Subharti Medical College and Lala Lajpat Rai Memorial Medical (LLRM) College District women hospital, in Meerut, Uttar Pradesh), in the north; King Edward Memorial Hospital (KEM Hospital), Pune, and Mahatma Gandhi Institute of Medical Sciences (MGIMS), Wardha, in the west; Jawaharlal Institute of Postgraduate Medical Education and Research (JIPMER), Puducherry, in the south; and Asian Institute of Public Health (AIPH), Bhubaneswar (with recruitments from SCB Medical College and Hospital, Cuttack, Capital Hospital, Bhubaneswar, and Rourkela Government Hospital, Rourkela), in the east. All six participant recruitment sites have a neonatologist as a study team member. The study will also include two institutions, namely, Vardhman Mahavir Medical College and Safdarjung Hospital (VMMC and SJH), New Delhi, and All India Institute of Medical Sciences (AIIMS), New Delhi, for the conduct of the gut colonization studies and quality control of the laboratory methods/procedures, respectively. As a pre-requisite for financial disbursal by the funding agency, the proposal has been reviewed by the Research Ethics Committee of Liverpool School of Tropical Medicine (REC-LSTM) that has provided a favorable opinion after an extensive review. The study has also received a favorable opinion from institutional ethics committees of all participating institutions and approval from the national regulatory authority (Central Drugs Standards Control Organisation, CDSCO), Government of India. The study has been registered with Clinical Trial Registry India (CTRI) with registration number: CTRI/2019/05/019197 (dated 16 May 2019).

### Study implementation and conduct

#### Study implementation

The study will be conducted in the following three phases.

I. *Preparatory phase*: This phase would cover the initial 6 months before recruitment of study participants. The tasks planned to be completed during this phase involve execution of a tripartite agreement between the sponsor and the pharmaceutical companies for supplies of investigational products (IPs), and a clinical trial agreement between the sponsor and the participating sites; constitution of a coordination committee with study staff from ICMR, International Clinical Epidemiology Network (INCLEN) Trust International, and MGIMS as its members and referred to as study collaborators (SC) to look after conduct of the trial; constitution of a trial steering committee (TSC) comprising of subject-matter experts as per guidelines of the funding agency; conduct of investigators’ meetings to complete the clinical trial-related tasks, namely, finalization of study protocol and standard operating procedures (SOPs) for all the study-related activities such as monitoring plans and visits, data collection processes along with their review and verification, safety reporting, etc.; constitution of a Data Monitoring Committee (DMC) to monitor ethics policies, protection of rights of participants, management of adverse events, etc.; laboratory set-up and SOPs for blood collection, culture and sepsis screen; applications to Ethics Committees (ECs) of respective sites and to CDSCO, India, for the approval to conduct the study; recruitment of support staff and their training; finalization of study tools and software, its installation, and training; insurance policy for the trial; supply of IPs and other study-related requirements to sites and hiring of a contract research organization (CRO) for site readiness check (list of site investigators and site personnel, adequacy of resources and facilities, documentation of protocol, SOPs, approvals, and other essential documents in trial master file, staff training, etc.).
II. *Intervention phase*: This phase would last for 18 months and would start with a site-initiation visit by study collaborator team member/CRO for initiation of enrolment of eligible infants in the study. The follow-up of enrolled infants would take place as per a pre-specified schedule for recording morbidities and outcome assessments at the six participating sites. Screening for morbidities would be conducted by trained field workers in the community, and sick infants would be referred to designated clinics/hospitals. A physician would examine the referred infants presenting with complaints and clinical signs, and blood samples would be collected from sick infants for diagnosis of neonatal sepsis. Stool samples would be collected from a randomly selected subset of enrolled infants at LHMC, New Delhi, on days 0, 21, and 60 of follow-up for gut colonization studies.
III. *Data analysis and reporting phase*: This phase of 6-month duration will start after the desired sample size has been achieved and follow-up of the enrolled infants is completed, and data from the participating sites has been verified. The final analysis would be conducted in a blinded manner as per the statistical analysis plan (SAP), and the codes will be broken after DMC permission. Drafting of study reports and dissemination of results would be done for concerned stakeholders including regulators, scientists, academia, and policy-makers. The study manuscript would be drafted followed by its critical review and finalization by all study investigators. The manuscript would be submitted to a peer-reviewed journal for publication irrespective of positive or negative results by the sponsor after approval from study investigators.

#### Study conduct

##### Informed consent process and enrolment

Healthy LBW infants need to be screened and enrolled within the first week of life, and therefore, efforts would primarily target postnatal women in participating sites. Biological parents of eligible infants will be informed about the study by study physicians. They will be enquired regarding their willingness for their baby’s participation in the study, and either parent will be asked to sign/put thumb impression (if illiterate) on the informed consent document on expressing approval for inclusion of their baby in the study. The parents will be informed about the visit schedules, administration of IPs, and collection of infant blood and stool sample. Informed consent would be taken based on regulatory guidelines and community norms. Enrolment will be done by study physicians at each site.

##### Follow-up visits

Follow-up visits in the community will be done by field workers who would visit daily in the first week of life, thrice per week during weeks 2–4 of life, and weekly once in the second month of life. Field workers will be trained to screen and detect sick infants; in case of any complaints, the field worker will accompany the parents with their infant(s) to the site hospital for further examination by the study investigator. Sepsis screen and blood culture would be performed if considered necessary, and appropriate treatment would be provided as per hospital or study protocol.

**Fig. 1:**
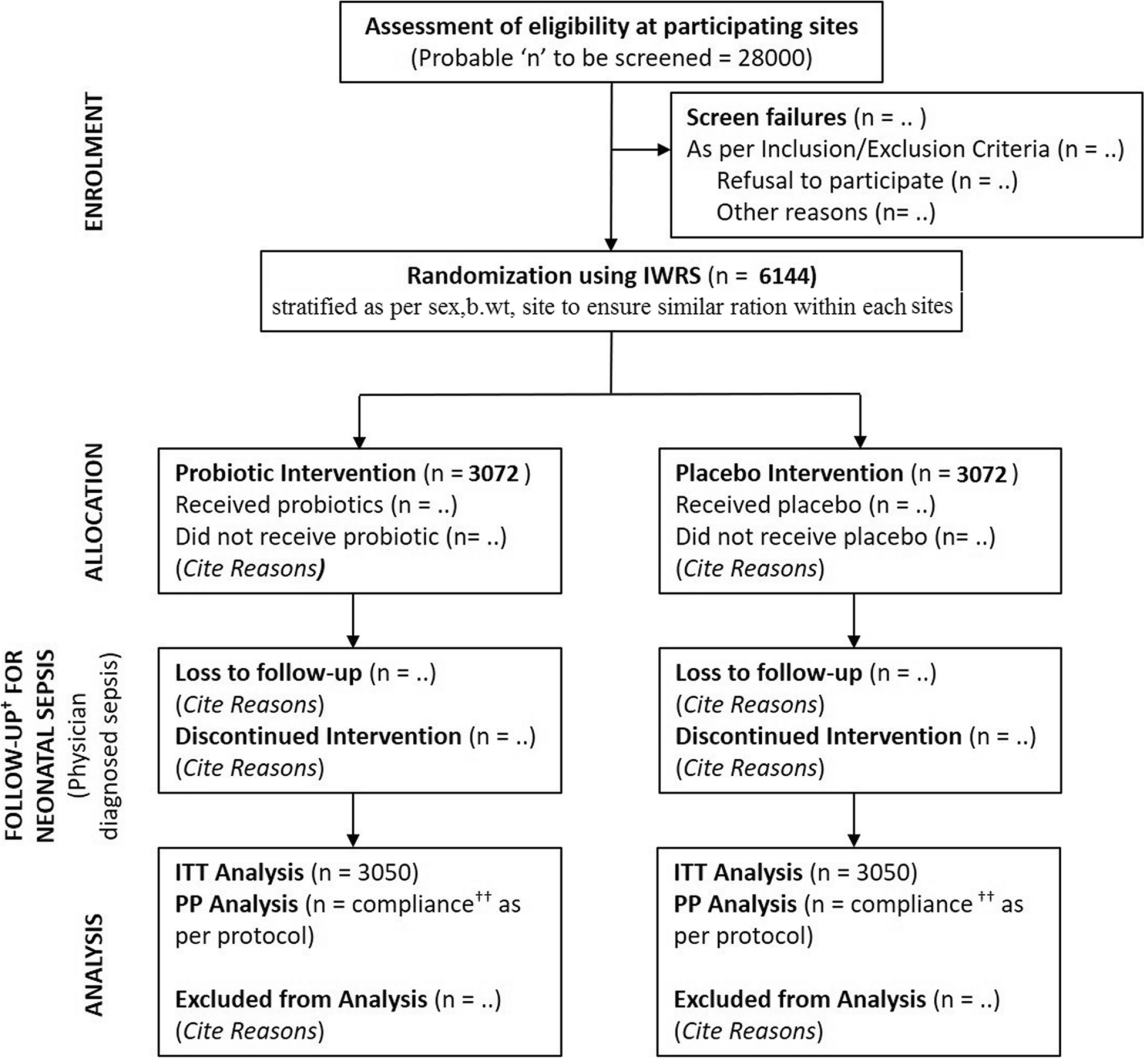
Study Design. Study design randomization stratification by sex, birth weight, and study site. ITT, intention-to-treat; PP, per-protocol. One dagger indicates daily during the first week of life, thrice in weeks 2–4 of life, and subsequently weekly in the second month. Two daggers indicate compliance defined as those enrolled infants who have ingested the study drug (for at least 25 days) and were followed up for more than 50% of scheduled visits

A schematic overview of the trial design is provided in Fig. 1, and an overview of the timeline of study intervention and assessments is provided in Table 1. The following measures will be adopted for obtaining a high level of compliance with regard to usage of the IPs in the study, namely, first-time demonstration and administration of IP would be done by the study investigator, and subsequent administration of IP would be done under the supervision of field worker on visit days during the first week, training of parents on the mode of administration, regular monitoring of contents in the pack and back-collection of empty bottles during field-worker visits, and filling up of a compliance form with date and time of administration of IPs during field-worker visits, and by either parent on other days.

**Table 1:**
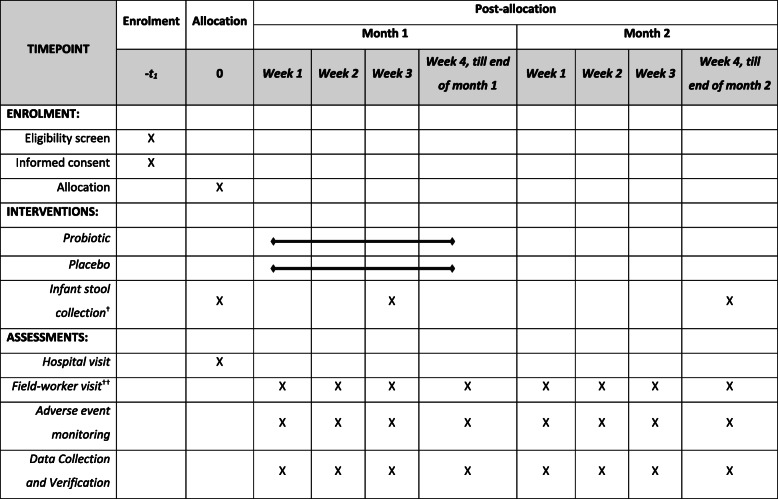
Schedules for study subject enrolment, intervention, and assessments

^†^Infant stool samples collected from one participating site (preferably in Delhi) at time of enrolment (day 0), at end of 3rd week of supplementation (day 21), and at end of follow-up (within day 56–60)

^††^Daily during the first week of life, thrice a week from week 2 of life till the end of the first month, and subsequently weekly in the second month

##### Study monitoring

The CRO and SC or their authorized representatives will inspect the participating sites for resources, facilities, trial master file (study protocol and supporting documents, study approvals), and study binders for participants, as well as observe the readiness of sites (availability of participants, IP availability, staff training, tools, and software) for the recruitment process. The CRO and the SC will also be responsible for regular site monitoring visits and for inspecting the trial records (informed consent documents, source data verification, trial records, safety reporting). Monitoring will be conducted according to CRO’s SOPs and study-specific monitoring plan. CRO will assess adherence to the protocol/SOPs and confirm the quality and accuracy of data collected at the study site, including the validation of data collected on tablets (e-forms), site-specific availability and reconciliation of IPs; raise queries; and assess the resolution of any past queries identified during previous monitoring visits. Protection of rights of human participants and compliance as per research guidelines and regulations, including procedures for ensuring confidentiality, informed consent, and regulatory documentation, will be of utmost concern and will be monitored. Safety assessments and reporting will be performed together by site investigators, CRO, sponsor, and DMC as per regulatory guidelines.

### Eligibility of study participants

#### Inclusion criteria

- Infant birth weight ≥ 1500 to ≤2500 g
- Age of the infant on recruitment falls between 3rd and 7th day of life
- Stable clinical condition as assessed by the physician (“stable” means one who does not require intravenous fluids and vasopressor medication to maintain circulation and accepts oral feeding/breastfeeding)
- The mother (with the infant) is planning to stay in the study area (residing within 30 km), for at least 2 months

Exclusion criteria

- An infant with extreme prematurity (< 34 weeks)
- An infant with illness requiring prolonged hospitalization and interference with oral feeds
- Presence of a gross congenital malformation incompatible with life
- Parent/legal authorized representative (LAR) not providing written consent

### Intervention and laboratory assessments

#### Intervention

The probiotic preparation contains a mix of 8 strains of bacteria, namely, *Lactobacillus plantarum* DSM 24730® / NCIMB 30437, *L. paracasei* DSM 24733® / NCIMB 30439, *L. delbrueckii* subsp. *bulgaricus* DSM 24734® / NCIMB 30440**, *L. acidophilus* DSM 24735® / NCIMB 30442, *Bifidobacterium longum* DSM 24736,® / NCIMB 30435*, *B. infantis* DSM 24737® / NCIMB 30436*, *B. breve* DSM 24732® / NCIMB 30441, and *S. thermophilus* DSM 24731® / NCIMB 30438 (* Re-classifié comme B. lactis** Re-classifié comme L. helveticus), suspended in medium-chain triglycerides (MCT) oil. The investigational product, probiotic mix, has been registered with the Central Drugs Standard Control Organisation CDSCO, India, and is commercialized/available under the brand Vivomixx® [23] in Europe, as Visbiome® in the USA, and as DeSimone Formulation in Korea. The dosage form of the suspension is standardized to deliver 10 billion CFU/ml, on reconstitution with MCT oil. Placebo contains maltodextrin instead of the probiotic blend that needs to be reconstituted in a similar way. The packs are identical in appearance, taste, and smell. The recommended dose for usage in the study will be 1 ml daily for 30 days.

The IPs will be supplied in a novel packaging, containing a vial (with 5 ml of MCT oil), a tap (containing at least 50 billion CFU of probiotic bacteria) fit on the top of the vial, and an endcap to seal the contents. During usage, the endcap needs to be screwed clockwise to obtain complete rupture of the ring nut and for release of the tap contents into the bottle that needs to be shaken vigorously to obtain a uniform suspension. The endcap (with tap) should be removed and replaced with a drop dispenser. The reconstituted product should be shaken before every use and one dropper should be used for each bottle. The product should be stored refrigerated at 2–8 °C and should be used within 3 weeks after reconstitution.

#### Laboratory support for blood culture, sepsis screen, and gut colonization assessment

Methodology for sepsis screen and BACTEC blood culture will be developed and standardized at AIIMS. Facilities for sepsis screen will be developed at the main hospital at each participating site, and a designated, appropriately trained person in the accredited laboratory will be responsible for performing the tests. Uniform centralized training of laboratory technicians from all study sites will be undertaken at AIIMS, New Delhi, which would function as the quality assurance laboratory. Gut colonization study will be conducted at VMMC & SJH on samples from only one participating site (LHMC, New Delhi).

#### Randomization, allocation, and blinding

Randomization codes will be generated and provided to the six sites through an interactive web response system (IWRS) using a standard web browser and email service. A 1:1 ratio will be used in randomization that will be stratified by the participating site, sex, and birth weight (1500–2000 g; 2001–2500 g) to ensure a similar ratio within each site. Randomization shall follow variable blocks of sizes 4 and 6. The IWRS system will also assign treatment allocation, provide automated confirmation of assignment (on-screen and via email), and provide regular reports on trial progress. The selection of subjects at one participating site for stool collection and gut colonization study will also be done using the IWRS system.

The site investigators, study staff (including field workers, study coordinators, etc.), and the parents of participating infants will be masked about the allocation of study participants to either intervention or placebo group. A pack containing the IP bearing the same enrolment number as that of the randomized infant shall be used for that participant.

### Study outcome measures

#### Primary outcome measures

**Fig. 2:**
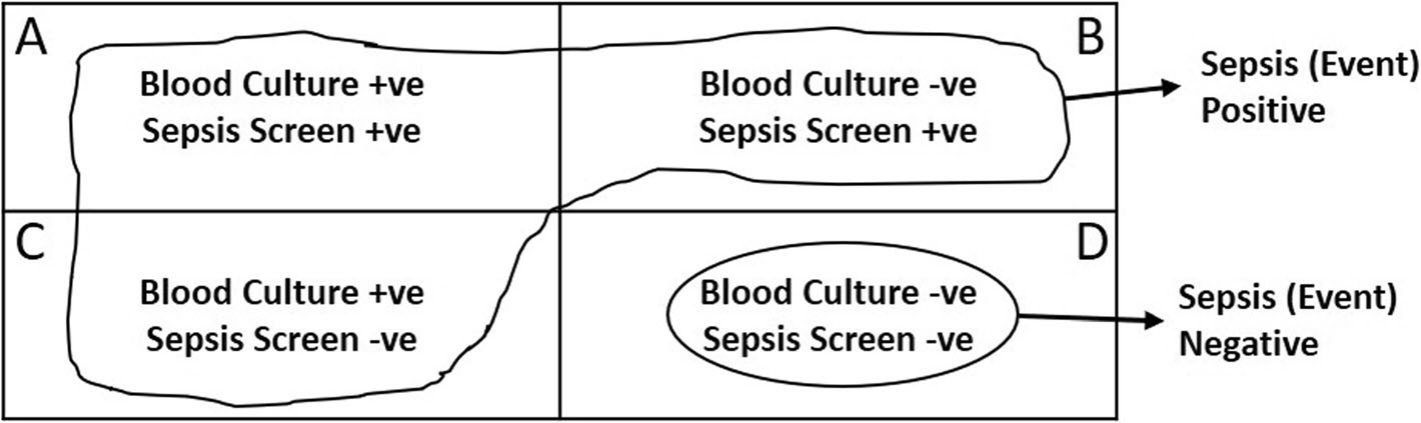
Possible outcomes of sepsis screen and blood culture

- *Sepsis* (defined as with one or more clinical signs suggestive of sepsis with a microbial isolate on blood culture or a neonate with sterile blood culture with at least 2 sepsis screen markers being abnormal (C-reactive protein > 12 mg/dl, absolute neutrophil count < 1500/mm^3^, total leucocyte count < 5000/mm^3^, erythrocyte sedimentation rate > 15 mm, immature to total neutrophil ratio > 0.2))
  Figure 2 shows the possible outcomes of the sepsis screen and blood culture tests performed on sick infants. The outcomes as shown in cells A, B, and C will be considered as an outcome event of interest (sepsis-positive) for the study. A sick infant with negative sepsis screen and negative blood culture in cell D (true negative) will not be considered as sepsis and will not contribute to the outcome event of interest.
  Other investigations such as X-ray chest and culture for cerebrospinal/peritoneal/pericardial/pleural fluid will not be mandatory for the diagnosis of sepsis in the study; however, these tests would be done based on-site investigator’s judgment, and as per the clinical situation.
- *Possible serious bacterial infection* [24] (*PSBI*) {defined as with one or more clinical signs, namely, not feeding well, convulsions, severe chest in-drawing, fever (temperature ≥ 38 °C), low body temperature (< 35.5 °C), movement only when stimulated or no movement at all, and fast breathing (60 breaths per minute or more) in infants less than 7 days old, as confirmed by study physician}

#### Secondary outcome measures

- Stool colonization patterns at baseline (day 0), day 21, and at the end of the study (within day 56–60) in a sub-sample of recruited participants
- Death and late-onset sepsis
- Clinical severe infection {defined as with one or more clinical signs, namely, not feeding well, fever (temperature ≥ 38 °C), low body temperature (< 35.5 °C), severe chest in-drawing, and the movement only when stimulated, as confirmed by the study physician}
- Critical illness {defined as with one or more of clinical signs, namely, convulsions, unable to feed at all, no movement on stimulation, unable to cry, bulging fontanelle, and cyanosis as confirmed by the study physician}
- Cost-effectiveness of the probiotic intervention vs standard care (proposed to be taken up as a sub-study)

### Withdrawal of participants

Participants shall have the right to withdraw from the study at any time for any reason; however, efforts would be made to prevent such instances. The reasons for discontinuation will be recorded in the participants’ study file. Any identified adverse events will be followed until resolution. Non-compliance with the study protocol, significant protocol deviation, any serious adverse events potentially related to study treatment, etc. would be recorded systematically for discussions with DMC and appropriately resolved.

### Data collection and management

The INCLEN Trust International, New Delhi, will supervise data collection and data management. Computer-assisted personal interview (CAPI) software on tablets with fast/real-time transmission of the data to the INCLEN server will be provided to site investigators and field staff. Study-related information will be collected on structured formats created using software with an inbuilt system for step-by-step data collection, checking, and verification process to allow rapid data review and rectification.

Data will be synchronized into the INCLEN server by the study data manager after preliminary cleaning preferably within 24 h of collection of participant information. The study data in the INCLEN server shall be checked and verified by the data management team regularly. The data shall be stored in the database after verification. Authorized personnel with strict security systems will have access to data. Logs for access and modifications in data shall be monitored through the data management protocol. Procedures to assure confidentiality will be strictly observed and the data collected shall be treated as confidential.

### Sample size and statistical analytical plan (SAP)

#### Sample size for the trial and the gut colonization study

The sample size for this two-arm parallel design superiority trial has been calculated based on the results of the earlier study with the same intervention in LBW babies [22]. The proportion of physician-diagnosed sepsis was reported to be 0.082 in the placebo arm as against 0.057 in the probiotic arm. Considering the relative reduction of 30% in the probiotic arm as compared to the placebo arm, with 5% type I error, 90% power, and 10% margin of superiority, the sample size required in each arm was calculated as 2764. Assuming an attrition rate of 10%, the sample size required in each arm was revised to enroll at least 3072 infants. Hence, a total of about 6144 LBW infants are proposed to be enrolled in the trial to have meaningful evidence on the superiority of the proposed intervention.

Based on the findings of the interim analyses for the first 1000 participants with 2-month follow-up, the sample size will be revised after consultation with the TSC members, if necessary. Sample size recalculation will be done based on the incidence of physician-diagnosed sepsis; however, the margin of superiority and all other assumptions would remain the same. If needed, additional sites would be identified and included in the study.

The sample size calculation for the gut colonization study has been calculated to measure a difference of 15% in the proportion of study participants with colonization by intervention probiotic as compared to the placebo arm. After adjusting for a 15% inadequate number of samples, the sample size is proposed for 200 study participants in each arm with analysis at three-time points for each participant.

#### SAP

The analysis would be done on pooled data from the study sites. The INCLEN Data Management Team shall develop an SAP that would be reviewed and approved by the Data Monitoring Committee, before data processing and analysis. Data analysis will be done using Stata 15.0 and R version 3.4.0 or above. The data analysis shall follow RCT guidelines and would be performed as per intention-to-treat and per-protocol analysis. The routine analysis would be by intention-to-treat. Data on non-compliers, protocol violators, and study drop-outs would be handled in a way such that participants assigned to the intervention arm will be considered as belonging to that arm even if he/she has not complied with the study protocol. A separate per-protocol analysis will also be done including only those cases who have complied with the intervention. Enrolled infants who have ingested the IPs for at least 25 days and were followed up for more than 50% of scheduled visits would be considered as compliant. A comparison of intervention and control groups will be done for the cumulative incidence ratio as well as the incidence density ratio. Survival analysis and hazard ratio estimation will be done separately for primary or secondary outcome considering the first episode of the outcome as the event.

## Discussion

This adequately powered and well-designed pan India trial will be conducted at six geographically distributed locations to conclusively evaluate the efficacy of probiotics in the prevention of neonatal sepsis and to complement the results from our previously completed study by generating additional data on the safety and efficacy of the probiotic preparation in low birth weight infants. The dosage form has undergone a significant improvement after the last study where a sachet containing the probiotic bacterial strains was mixed with expressed breast milk before administration to the infant. This study proposes to use a new, standardized, and more convenient dosage form that is foolproof as far as contamination is concerned and may allow future up-scaling if the intervention proved to be efficacious based on the results of this trial. No changes have been done in the probiotic composition, its dose, and the duration of administration. Changes in the gut colonization pattern between the participants in probiotic and placebo arms were observed in the previous study; however, the changes were not significant [22]. Therefore, this study proposes to conduct the gut colonization studies in a larger sample size to substantiate the clinical results.

This study differs from a recently published study on the prevention of sepsis in neonates in India by Panigrahi et al. with regard to the product composition (probiotic vs synbiotic), the geographic distribution of trial sites, dosing regimen and mode of administration, inclusion criteria (enrolment of LBW infants between 3 and 7 days of life vs term infants enrolled within first 4 days of life), and probiotic bacteria strains used in the trial [21]. A systematic review and meta-analysis of 25 studies by Aceti et al. revealed that significantly fewer infants in the probiotic group (*p* < 0.001) developed LOS as compared to the control group (399 (13.60%) vs 506 (17.24%), respectively) [18]. Zhang et al. reported that probiotic supplementation was safe and effective in reducing the risk of LOS in preterm neonates in neonatal intensive care units [19]. Similarly, a systematic review of 37 RCTs (*N* = 9416) by Rao et al. [20] showed that probiotic supplementation resulted in a statistically significant decrease in the risk of LOS in preterm infants born at < 37 weeks or < 2500 g which were in contrast to other large previous meta-analyses, namely, Cochrane review [25] (*N* = 5338) and Lau et al. [26] (*N* = 5215), that did not find a statistically significant benefit of probiotic supplementation in reducing LOS in preterm infants. The Cochrane Review reported a “trend” towards a reduction in LOS with probiotic supplementation (RR 0.91; 95% CI, 0.80–1.03), but probably the sample size was insufficient to detect significance [25].

Deshpande et al. conducted a systematic review of randomized controlled trials (RCTs) from low- and medium-income countries (LMICs) on neonates (< 37 weeks) or LBW or both for all-cause mortality, LOS, and definite necrotizing enterocolitis (NEC) [27]. Data with definite NEC from 20 studies (*N* = 4022) showed that a higher proportion of neonates developed definite NEC in the control group than in the probiotic group (65/20165 (3.1%) vs 135/1957 (6.9%)) and meta-analysis using a fixed effects model (FEM) estimated a lower risk (RR 0.46 (95% CI 0.34 to 0.61), *p* < 0.00001) of NEC in the probiotic group. Data from 18 trials (*N* = 4062) revealed that a higher proportion of neonates developed LOS in the control group than in the probiotic group (308/2076 (14.5%) vs 358/1986 (18%)) and meta-analysis using a FEM estimated a lower risk (RR 0.80 (95% CI 0.71 to 0.91), *p* = 0.0009) of LOS in the probiotic group. Data from 19 trials (*N* = 4196) showed a reduced risk of all-cause mortality in the probiotic group than in the placebo group (137/2148 (6.37%) vs 176/2048 (8.59%)) and meta-analysis using a FEM estimated a lower risk (RR 0.73 (95% CI 059 to 0.90), *p* = 0.003) of death in the probiotic group.

Results from previous studies have revealed that probiotics are effective in significantly reducing the risk of all-cause mortality, LOS, and NEC in LBW neonates. Probiotics are generally considered to be safe (GRAS), and the lack of serious adverse events from multiple trials is encouraging. Probiotics are also routinely used in preterm neonates for the prevention of NEC in developed countries [28, 29]. Rare reports of sepsis in preterm newborns and bacteremia and endocarditis in adults potentially linked to probiotic administration have been observed [30–33]. However, the risk of developing bacteremia from ingested *Lactobacillus* probiotics is estimated to be less than one in a million [34]. The probiotic to be used in this study has been studied in over 60 RCTs including neonates [22] and infants [35, 36], and no serious adverse event has been reported.

The current literature does not provide a definite recommendation on which probiotic strain or mixture would be more effective in reducing NEC and sepsis in the vulnerable neonatal population. Different types of probiotics have been used in previous studies and many of them have shown a beneficial effect in this population, possibly through the development of a better ecological barrier and a better immunological profile. The target of under-five mortality under SDG can be achieved only if the neonatal mortality rate is reduced.

This current study would possibly be the single largest RCT and would answer the questions on the safety and efficacy of probiotics in the prevention of neonatal sepsis; however, the actions of individual probiotic strains or multistrain probiotic preparations are specific, and therefore, it would not be generalized to other probiotic formulations differing in species and strains, in the colony-forming units (CFUs), and in ratio/composition of strains. The study would emphasize on the government’s health policy “MAA” (Mothers Absolute Affection) of “No Interference with breastfeeding.” The single daily supplementation of probiotic drops will not interfere with exclusive breastfeeding policy and the study SOPs would also emphasize on the importance of exclusive breastfeeding. In case the product turns out to be efficacious and the study outcomes are positive, ICMR would facilitate a scale-up program for a greater benefit of the LBW infant population of the country and other developing countries.

## Data Availability

Data (protocol, data collection forms, the format of visit log, drug dispending log, temperature log, etc.) is available on request to researchers who provide a methodically sound proposal and whose proposed use of the data has been approved by an independent review committee identified for this purpose. Such proposals may be directed to the corresponding author (apradhandr@gmail.com). However, to gain access, data requestors will need to sign and submit a cover letter mentioning the purpose with a list of requested documents along with a statement/undertaking to maintain data confidentiality.

## Abbreviations

AIIMS: All India Institute of Medical Sciences
AIPH: Asian Institute of Public Health
CAPI: Computer-assisted personal interview
CDSCO: Central Drugs Standards Control Organisation
CFU: Colony-forming unit
CPHK: Center for Public Health Kinetics
CRO: Contract research organization
CTRI: Clinical Trial Registry India
DMC: Data Monitoring Committee
EC: Ethics Committee
ICMR: Indian Council of Medical Research
IgA: Immunoglobulin A
INCLEN: The International Clinical Epidemiology Network
IPs: Investigational products
IWRS: Interactive web response system
JIPMER: Jawaharlal Institute of Postgraduate Medical Education and Research
KSCH: Kalawati Saran Children’s Hospital
LAR: Legally authorized representative
LBW: Low birth weight
MCT: Medium-chain triglycerides
MGIMS: Mahatma Gandhi Institute of Medical Sciences
NEC: Necrotizing enterocolitis
PSBI: Potential suspected bacterial sepsis
RBM&CH: Reproductive Biology, Maternal and Child Health
RCT: Randomized controlled trial
SC: Study collaborators
SAP: Statistical analysis plan
SDG: Sustainable Development Goal
SOPs: Standard operating procedures
TSC: Trial Steering Committee
VLBW: Very low birth weight
VMMC & SJH: Vardhman Mahavir Medical College and Safdarjung Hospital
WHO: World Health Organization
